# Safety, Effectiveness and Feasibility of Outpatient Management of Children with Pneumonia with Chest Indrawing at Port Moresby General Hospital, Papua New Guinea

**DOI:** 10.1093/tropej/fmy013

**Published:** 2018-04-06

**Authors:** Rose Morre, Kone Sobi, Wendy Pameh, Paulus Ripa, John D Vince, Trevor Duke

**Affiliations:** 1 Department of Paediatrics, Port Moresby General Hospital, Port Moresby, NCD, Papua New Guinea; 2 Mt Hagen General Hospital, Mt Hagen, WHP, Papua New Guinea; 3 Child Health Discipline, School of Medicine and Health Sciences, University of PNG, Taurama Campus, Port Moresby, NCD, Papua New Guinea; 4 Centre for International Child Health, University of Melbourne, MCRI, Parkville, Victoria, Australia

**Keywords:** pneumonia, pneumonia with chest indrawing, home care, WHO recommendations

## Abstract

Implementing the World Health Organization (WHO) recommendations on home-based management of pneumonia with chest indrawing is challenging in many settings. In Papua New Guinea, 120 children presenting with the WHO definition of pneumonia were screened for danger signs, comorbidities and hypoxaemia using pulse oximetry; 117 were appropriate for home care. We taught mothers about danger signs and when to return, using structured teaching materials and a video. The children were given a single dose of intramuscular benzylpenicillin, then sent home on oral amoxicillin for 5 days, with follow-up at Days 2 and 6. During the course of treatment, five (4%) of the 117 children were admitted and 15 (13%) were lost to follow-up. There were no deaths. Treating children with pneumonia with chest indrawing but no danger signs is feasible as long as safeguards are in place—excluding high-risk patients, checking for danger signs and hypoxemia and providing education for mothers and follow-up.

## INTRODUCTION

Acute lower respiratory infections (which include bacterial pneumonia and viral bronchiolitis) are the commonest cause of hospital admission in most low- and middle-income countries. In recent years, trials in several countries have demonstrated the safety of home treatment for pneumonia with chest indrawing (previously classified by the World Health Organization [WHO] as severe pneumonia, [1–4]). The WHO has promoted the community case management of pneumonia with chest indrawing [5, 6]. Application of this policy is challenging. Many countries do not have strong outpatient services or adequate follow-up mechanisms to ensure the safety of this management. In the published trials, there were many exclusion criteria, meaning that safety and efficacy are demonstrated only in a selected population of low-risk children. Such case selection is not always easy in a children’s outpatient department. Highly supervised trials can ensure parents are properly educated on danger signs to look for and when to re-attend, but implementation in a real-world setting requires a deliberate strategy for education and safeguards. In Papua New Guinea (PNG), the predominance of bacterial pneumonia and its high mortality, the low education status of mothers and health systems that have focused on hospitalization rather than outpatient care have meant that outpatient management of pneumonia with chest indrawing has not been widely adopted. However, with increased demand for hospital beds, many affected children are sent home, without guidelines or approach to ensure safety and follow-up. This study was designed to trial a model of outpatient management of children with pneumonia with chest indrawing and to determine its safety, efficacy and feasibility.

## METHODOLOGY

This study was conducted in the Children’s Outpatient Department in Port Moresby General Hospital. Children of age 1 month to 12 years were eligible if presenting with signs and symptoms of pneumonia with chest indrawing—previously classified as severe pneumonia by the WHO and classified as moderate pneumonia in the PNG guidelines (Table 1).

**Table 1:** PNG standard classification of pneumonia and treatment implications before this study [8]

Pneumonia classification: PNG (WHO equivalent)		
Cough and difficult breathing plus …		
Classification	Signs	Treatment
Severe pneumonia (severe pneumonia)	Danger signs or hypoxaemia (SpO_2_ <90%) or cyanosis	Admit, give oxygen, benzyl penicillin (or ampicillin) and gentamicin intravenous
		If cough persist more than 14 days, assess for tuberculosis
Moderate pneumonia^a^ (pneumonia with chest indrawing)	Chest indrawing, but no danger signs or hypoxaemia	Admit, benzylpenicillin intravenous for 24 h; if improved, then change to amoxicillin for 5 days
		If cough persist more than 14 days. assess for tuberculosis
Mild pneumonia (pneumonia)	Fast breathing, but no chest indrawing, danger signs or hypoxaemia	Home on oral amoxicillin
Simple cough	Normal respiratory rate, no chest indrawing and no danger signs	Home with symptomatic treatment only (maintain oral fluids, continue breast feeding, e.g. paracetamol if febrile)

a Equivalent to current WHO classification of pneumonia with chest indrawing.

The following characteristics were screened for, using history and examination, and if found, children were excluded and managed in alternative ways (primarily as inpatients):

- chronic illnesses, including severe malnutrition, tuberculosis, anaemia, HIV, asthma or chronic lung disease;
- danger signs, including severe respiratory distress, cyanosis, inability to drink, hypoxaemia as measured by pulse oximetry, signs of shock, heart failure (hepatomegaly and heart rate >160) and convulsions [7];
- vomiting all feeds or medicine, which would reduce the effectiveness of oral therapy; and
- home location and transport access for feasibility of follow-up.

Clinical information recorded included duration of cough, adequacy of feeding, presence of vomiting, convulsion or any danger signs, heart rate, respiratory rate, axillary temperature, presence of wheeze, chest indrawing and oxygen saturation using a hand-held pulse oximeter.

Mothers and fathers were shown a short video on recognizing danger signs. These included severe respiratory distress, head nodding, cyanosis, poor feeding and convulsions. The mothers’ understanding of these signs was assessed, and their ability and willingness to return for follow-up were confirmed.

After assessment, children were given a stat dose of benzylpenicillin, 50 000 international units per kg, by intramuscular injection. They were observed for 2–4 h to ensure their SpO_2_ was >90% in air, there were no danger signs and parents understood how to give home treatment, what to monitor for and when to return. The mothers were taught to give oral amoxicillin to the children (how much, how often and cleanliness). Children went home on oral amoxicillin 25 mg/kg every 8 h for 5 days. They were asked to come back the following day for review.

### Follow-up

On Day 2, COPD staff identified children on outpatient treatment by a yellow sticker on their clinic books. The mothers were interviewed regarding the child’s condition, administration of amoxicillin and whether the child had any danger signs. Vital signs and SpO_2_ were recorded, and a clinical examination completed. If no danger signs or hypoxaemia had developed, they were sent home to complete the amoxicillin course for a total of 5 days. They were requested to return on Day 6 for the final review. If at any review the children had developed danger signs or hypoxaemia or if on Day 6 they remained unwell, they were admitted to the ward, re-assessed and treated accordingly.

Children who did not come for review on Day 2 and Day 6 were followed up by telephone calls to assess if they were improving and to understand reason for non-attendance.

Treatment failure was defined as deterioration in the child’s condition necessitating admission to hospital, if signs of pneumonia with chest indrawing persisted on Day 6 or if the child died at any time during the study or in the month from enrolment.

Safety was assessed by recording admission rates and deaths. Effectiveness was assessed by assessing reduction in symptoms and signs at Day 2 and their resolution by Day 6. Feasibility was assessed by the ability of parents to recognize danger signs after having watched a teaching video, the proportion of children in whom oximetry was possible and reliable in an outpatient setting and the proportion of mothers who could demonstrate competency in administering amoxicillin.

Data were entered into an Excel spreadsheet and analysed using STATA version 14. The study was approved by the University of Papua New Guinea Research Committee and the Director Medical Services of Port Moresby General Hospital. Consent was obtained from parents or guardians before inclusion in the study.

## RESULTS

**Fig. 1.**
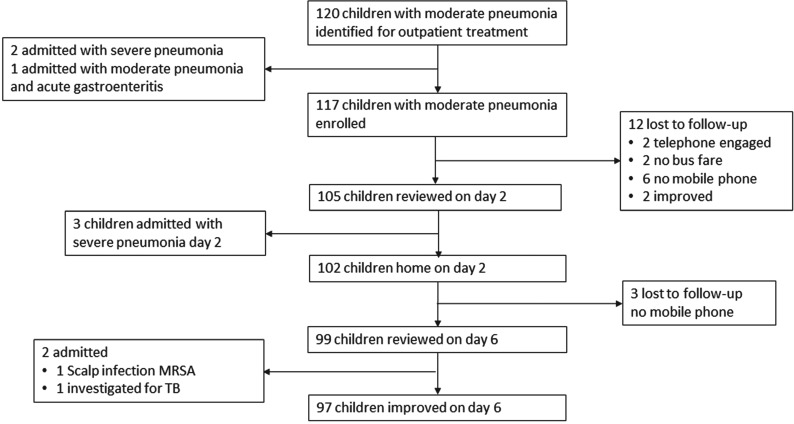
Flowchart summary of the study.

Children were enrolled between July 2015 and July 2016 (Fig. 1). After initial screening, 120 children were identified with moderate pneumonia. Of which, 49 (40%) were female. The median duration of cough was 3 days (interquartile range [IQR] = 1–5 days). Their median age was 12 (IQR = 6–24) months and weight was 8.95 (7.4–12) kg. In all, 67% of the mothers had some form of education (27% primary, 25% secondary and 15% tertiary), whereas 33% had no formal education. A total of 75 mothers (63%) had a mobile phone.

During the initial observation, two children developed danger signs and SpO_2_ was <90%, and one had diarrhoea as well as moderate pneumonia; these three children were admitted. In all, 117 children were sent home on oral amoxicillin.

On Day 2, 105 children returned for review; 102 were clinically stable or improved from the previous day (Table 2). Respiratory rate, chest indrawing, heart rate and wheeze had substantially reduced. Three had hypoxaemia and danger signs consistent with severe pneumonia and were admitted. Twelve children were not brought for follow-up on Day 2; reasons are given in Fig. 1.

**Table 2:** Clinical characteristics of the children with pneumonia with indrawing at baseline and Day 2

Clinical characteristics	Day 1 (baseline), *n* = 117	Day 2, *n* = 105
Respiratory rate: mean (SD)	52 (9.9)	39 (8.6)
RR 60 or more	17 (15%)	3 (3)
Heart rate	145 (25)	124 (22)
Heart rate 160 or more	33 (28%)	0
Temperature	37.7 (1.1)	37.7 (1.1)
Temperature >37.9°C	30 (26%)	3 (3%)
Oxygen saturation	97 (2.2)	97 (2.5)
Oxygen saturation <90%	0^a^	3^b^
Cyanosis	0	1
Chest indrawing	120 (100%)	54 (51%)
Wheezes	79 (65.8%)	28 (26.7%)
No feeding well	4 (3.3%)	4 (3.8%)

a Two children of the 120 enrolled developed hypoxaemia during the 2-h observation and were admitted; one had coexistent gastroenteritis and was admitted. Therefore none of the 117 who were treated as outpatients had hypoxaemia on day 1.

b On Day 2, three children had hypoxaemia and danger signs and were admitted.

On Day 6, 99 children were brought for review. Signs of ALRI had resolved in 97 children. Two children were admitted: one for a scalp infection (MRSA), and one because of persistent weight loss, who was investigated for pulmonary and lymph node tuberculosis. At Day 6, three children were lost to follow-up with no mobile phone contact.

Of the 117 children enrolled, five (4%) were admitted. There were no deaths. Of the 102 patients who completed follow-up, 97 (95%) had improved substantially on Day 6 after completion of 5 days of amoxicillin.

The protocol was feasible; after being shown the video, all parents were able to recall danger signs on Day 1 and Day 2, including not feeding, vomiting feeds, head nodding and cyanosis. Pulse oximetry was useful in detecting hypoxemia and screening children for suitability for outpatient treatment, and it helped parents understand that their child was being carefully monitored. With patience we were able to get pulse oximeter readings for all the children. On Day 2 and Day 6, 98 and 97 mothers, respectively, demonstrated that they could administer amoxicillin correctly, give the correct dose and understand the timing and cleanliness. Five and two mothers on Day 2 and Day 6, respectively, had received amoxicillin but had administered a lower dose than prescribed; however, their infants were not unwell. Two mothers on Day 2 had not given the antibiotic at all.

## DISCUSSION

In recent years, the WHO has promoted the community case management of pneumonia with chest indrawing but no other danger signs [5, 6]. This was previously classified by the WHO as ‘severe pneumonia’, distinct from a ‘very severe pneumonia’, in which the child has danger signs or hypoxaemia and requires hospital admission. Recently, the WHO classification has changed the term of ‘severe pneumonia’ to ‘pneumonia with chest indrawing’ to promote outpatient management [7].

In PNG, pneumonia classification has remained the same since the 1970s: mild, moderate and severe pneumonia (Table 1) [8]. The WHO recommendation to manage this type of pneumonia at home is based on large trials, mostly in urban and peri-urban areas of Pakistan, but also in Bangladesh, Egypt, Ghana and Vietnam [1–4]. However, the implementation of this recommendation in countries with a high burden of bacterial pneumonia, malnutrition and HIV and in populations with lower levels of maternal literacy and less accessibility to health services is challenging.

Children with clinical signs of ‘pneumonia with chest indrawing’, a syndromic diagnosis, may have a variety of conditions: from acute viral bronchiolitis to bacterial pneumonia. There is also a range of severity even within the clinical syndrome of ‘chest-indrawing pneumonia’; in PNG, many children with the same clinical features have hypoxaemia [9, 10]. Furthermore, without the assessment for comorbidities (malnutrition, anaemia, asthma and HIV), high-risk children may be discharged to home and die unexpectedly.

We aimed to evaluate the WHO recommendation on home treatment using a protocol that identified high-risk children and was safe. The safeguards included:

- excluding high-risk patients (children with HIV, severe malnutrition, or hypoxaemia and neonates);
- checking for danger signs and hypoxaemia with pulse oximetry;
- a protocol for education of mothers, including teaching about danger signs and when to return (using structured teaching materials and video) and teaching about how to give home antibiotic treatment; and
- follow-up assessment on Day 2 and Day 6, including pulse oximetry to detect hypoxaemia.

The rate of admission was low (five of the 102 for whom the outcome was known), and the rate of loss to follow-up (15/117, 13%) was also relatively low. Treatment success with outpatient management occurred in 97 (95%) of the 102 children with complete follow-up, and there were no adverse events and no known deaths.

Outpatient treatment of common moderate illnesses, including low-risk pneumonia or gastroenteritis, uses resources rationally and avoids exposing children to the risks of hospitalization. It saves health money, avoids hospital overcrowding and avoids families being separated with the primary caregiver away from other children. It can empower parents and teach them about care-seeking. If mothers need to stay in hospital while their child is unwell, it can be very difficult if they also have to care for other children. On the other hand, it is important to assess the ability of parents to return for follow-up, and it is important to ensure that the family’s home environment is conducive to home care of an ill child.

Reassessment of the diagnosis if a child is not improving is very important. Even children with chest indrawing pneumonia may develop complications such as hypoxaemia, empyema or sepsis.

The limited educational background of many of the mothers in this study did not seem to affect the success of the approach to educating them about danger signs, the importance of follow-up and the need for correct administration of amoxicillin.

Other outpatient pneumonia studies have not considered administering a dose of benzylpenicillin. The reasons we chose to do so were concern that mothers may not administer oral amoxicillin well, the high burden of bacterial infection in PNG and the high level of cultural acceptance of injections when a child is unwell. Mothers did not have to make a decision between injection and being admitted. The stat dose of benzylpenicillin may not be necessary, but only a large controlled trial would resolve this.

The WHO recommends amoxicillin twice daily at a dose of at least 40 mg/kg BD. This is similar to the daily dose we used in this study (25 mg TDS), which has been the standard dose in PNG for decades. In other studies, twice-daily amoxicillin has been associated with improved adherence, so this remains an option for outpatient treatment [5, 11]. We did not have dispersible amoxicillin, which is now recommended by the WHO [5].

In the case of *Haemophilus influenzae* type b (Hib) pneumonia, amoxicillin at any dose is not guaranteed to be effective because of high rates of β-lactamase production and β-lactamase-negative ampicillin-resistant strains. The rates of Hib vaccination in PNG provide protection for most children, but non-typeable *H. influenzae* has always been a problem in PNG. Rates of β-lactamase production and β-lactamase-negative ampicillin-resistant strains vary throughout the Western Pacific region, from very low levels reported in China to 30% in Vietnam [12, 13].

If children fail outpatient treatment, they need to be identified early; investigated with blood culture, full blood examination and procalcitonin test, if available; and treated with an antibiotic that covers resistant *Haemophilus* and pneumococcal pneumonia and other pathogens relevant to their clinical and environmental context. A common reason for ‘failing’ antibiotic therapy is the presence of a viral infection, mainly the respiratory syncytial virus, but also influenza, parainfluenza or adenovirus [14]. In the absence of microbiological testing, distinguishing persistent viral infection from a bacterial aetiology that has not responded to amoxicillin is difficult. Furthermore, co-infection—an infection that began as a viral LRTI and was complicated by a secondary bacterial infection—is common. If a child is unwell with pneumonia, with high fever, hypoxaemia or other signs of severe pneumonia having failed outpatient amoxicillin treatment, they should be treated with ceftriaxone or cefotaxime, or flucloxacillin and gentamicin, as per WHO guidelines for severe pneumonia, and have a chest x-ray to rule out complications, such as effusion [7].

Among children with ‘moderate pneumonia’ without hypoxaemia, many will be infants who only have acute viral bronchiolitis. For well children with fast breathing, mild chest indrawing, wheeze and a temperature below 38°C, there is a role for not giving antibiotics at all, and this would require the same degree of follow-up and education that we used in this study [7, 15].

## Acknowledgements

We thank the nurses in the Children's Emergency Department who helped care for these children and provide education to their mothers. We are grateful to the RE Ross Trust (Victoria, Australia) for funding support to child health training and research in PNG.
